# Measuring the Water Content in Wood Using Step-Heating Thermography and Speckle Patterns-Preliminary Results

**DOI:** 10.3390/s20010316

**Published:** 2020-01-06

**Authors:** Francisco J. Madruga, Stefano Sfarra, Stefano Perilli, Elena Pivarčiová, José M. López-Higuera

**Affiliations:** 1 Photonics Engineering Group, CIBER-BBN and IDIVAL, Universidad de Cantabria, Plaza de la Ciencia s/n, 39005 Santander, Cantabria, Spain; lopezhjm@unican.es; 2 Department of Industrial and Information Engineering and Economics (DIIIE), University of L’Aquila, Piazzale E. Pontieri, no. 1, I-67100 L’Aquila (AQ), Italy; stefano.sfarra@univaq.it (S.S.); stefano.perilli@graduate.univaq.it (S.P.); 3 Faculty of Environmental and Technology Manufacturing, Technical University in Zvolen, Ul. T.G. Masaryka 2174/24, 960 53 Zvolen, Slovakia; pivarciova@tuzvo.sk

**Keywords:** infrared thermography, speckle inspection, wooden structures, nondestructive evaluation, moisture, defect, heat and mass transfer

## Abstract

The relationship between wood and its degree of humidity is one of the most important aspects of its use in construction and restoration. The wood presents a behavior similar to a sponge, therefore, moisture is related to its expansion and contraction. The nondestructive evaluation (NDE) of the amount of moisture in wood materials allows to define, e.g., the restoration procedures of buildings or artworks. In this work, an integrated study of two non-contact techniques is presented. Infrared thermography (IRT) was able to retrieve thermal parameters of the wood related to the amount of water added to the samples, while the interference pattern generated by speckles was used to quantify the expansion and contraction of wood that can be related to the amount of water. In twenty-seven wooded samples, a known quantity of water was added in a controlled manner. By applying advanced image processing to thermograms and specklegrams, it was possible to determine fundamental values controlling both the absorption of water and the main thermophysical parameters that link the samples. On the one hand, results here shown should be considered preliminary because the experimental values obtained by IRT need to be optimized for low water contents introduced into the samples. On the other hand, speckle interferometry by applying an innovative procedure provided robust results for both high and low water contents.

## 1. Introduction

One of the most important aspects in the use of wood for construction and restoration is the relationship between the material and the moisture content [1]. Indeed, the wood is a hygroscopic material, which means that it works like a sponge gaining or losing moisture from the air according to the environmental conditions [2]. This gain and loss of moisture also cause the expansion or contraction of wood according to the change of humidity it undergoes in the course of time.

One of the most popular techniques able to detect moisture in wooden structures is the infrared thermography (IRT) [3,4,5,6,7,8,9,10,11,12,13,14,15,16,17,18].

During the first years of the twenty-first century, thermographic techniques were applied to the inspection of wooden buildings in North America [19]. The measurement of the amount of moisture or water content in a point of the analyzed zone provided information of the entire area.

IRT is a nondestructive testing (NDT) technique that has several advantages such as the speed of inspection, exclusives application fields, and possibility to post-process the thermograms acquired on advanced materials [20,21,22]. It is also contactless and safety for the operator. The main disadvantages are the homogeneous heating to provide on the surface under inspection, as well as the control of the various mechanisms of heat transfer or mechanical responses [23]. The IRT modalities can be divided into passive (the target is at a higher temperature than its environment in normal conditions) or active (energy input is required to generate thermal contrast). Conventional IRT usually uses an optical excitation. In addition, other thermal sources including eddy current, laser, microwave, and ultrasound, have been applied in the thermographic field [24]. Pulsed thermography (PT), stepped heating thermography (SHT), lock-in thermography (LT) (also called modulated thermography (MT)), are the most important approaches. In wooden materials, the SHT is the most used due to the thermophysical parameters.

This technique can be integrated into a large number of metrology applications using patterns of structured light (i.e., specklegrams) and sharing the advantages previously described [25].

Terahertz (THz) imaging and spectroscopy are among the promising NDT techniques as well [26,27,28]. Unfortunately, THz equipment is not available in our respective laboratories.

In the present work, the authors present a study based on thermographic and optical measurements implemented with preprocessing and post-acquisition procedures. The optical method can be considered as innovative for the field of application studied here.

## 2. Materials and Methods

**Figure 1:**
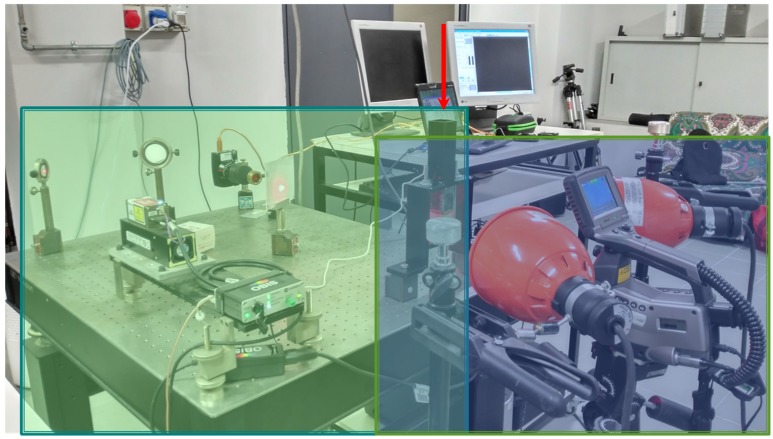
Combined experimental setup for thermographic and optical (speckle) measurements.

A dual speckle interference and an active thermography system implemented with a SH excitation were installed on a heavy-isolated stable table. The system was applied to the measurement of wood samples having different orientations of their natural fibers. The samples were supplied by the center of material of the Technical University of Zvolen in Slovakia. The green rectangle in Figure 1 illustrates the speckle inspection. It was put on the side of the sample holder. The dual system consisted of a red light laser of 660 nm (100 mW of power) and a system of mirrors that guide the light emitted. In addition, a frosted glass that provoked multi-directional guidance of the laser ending with a projection of a speckle pattern on the sample under analysis was added to the experimental setup [29]. A Canon SX monochrome camera (equipped with a bandpass filter centered at 660 nm and having a width of 10 nm) recorded every speckle pattern. On the opposite side, a FLIR S65 HS long-wave (LW) infrared camera (7.5–13 μm), along with two 250 W lamps, was installed on tripods to provide a homogeneous thermal excitation on the second sample surface inspected. Two computers, one for each camera-excitation set, controlled the entire experimental setup. The thermographic part is highlighted in blue on the right side of Figure 1.

Twenty-seven hexahedral parallelepiped-shaped specimens (20 × 20 × 50 mm) were inspected by the combined method mentioned above. The specimens are constituted of fibers following radial, tangential, and axial directions.

Each specimen was in a supposed dry state (weight = ~30 g). Specimens were placed, one by one, inside a support having at the top of a duct through which water was introduced. The red arrow clarifies the direction of the water ingress (Figure 1). The specimens fit perfectly inside the duct by avoiding the loss of water subjected to the gravitational force.

Three milliliters of water was added after each test until 3 cl in total was reached. The latter was considered the 100% water content.

Once the specimen was installed and the water was added, a total of 190 visible images were captured in a cycle based on the following steps:

- 10 visible images without any thermal excitation at work, (100s)
- 60 images with the laser activated, (600s)
- 50 images with the laser activated during heating-up phase, (500s)
- 70 images with the laser activated during cooling down phase (700s), and
- 130 thermograms (10 as background (100s), 50 during the heating-up phase (500s), and 70 during the cooling down phase (700s)).

The heating up and cooling down phases resembled a square pulse usually used in applications concerning cultural heritage and civil engineering [30]. Each thermogram was recorded every 10 s.

Readers should notice that, thanks to the frosted glass (Figure 1), the laser worked as an illumination (and not heating) system.

Once in contact with the top surface of the specimen, the action due to capillarity taken place. The velocity of the capillarity rises in a capillary of radius *r* when, as in our case, the gravitational acceleration is taken into account. It is given by Yata [31] and follows Equation (1):

(1) $$\frac{dh}{dt}=\frac{r\sigma cos\theta }{4h\eta }-\frac{r^{2}\rho g}{8\eta }$$

where *h* is the height (maximum capillary rise) [m], *t* is the time [s] from the start of the experiment, *σ* is the surface tension [N/m], *θ* is the contact angle, *ρ* is the density [Kg/m^3^], *g* is the gravitational acceleration [m/s^2^], and *η* is the viscosity.

Because the water firstly impregnated the whole top surface of the specimens (i.e., 400 mm^2^) by percolating into them thereafter following Equation (1), it is possible to say that, in our case, the depth’s impact on the water content measurements is related to the thickness (i.e., 20 mm) of every specimen at *z* = 0. The value decreases along the *z*-axis (i.e., along the height = 50 mm) according to the Comstock model [32], which described the permeability of softwoods on the basis of lumens connected with pits on their tangential surfaces on their tapered ends.

## 3. Results and Discussion

### 3.1. Thermography Method

The determination of the water content using IRT was based on the SH modality. The sample was heated for 500 s in each test, and a thermogram every 10 s was recorded. The sequence of cooling down phase lasted seven hundred seconds. The heating time was chosen according to References [19,33].

Applying the temperature evolution (T(*t*)) formulation on the hexahedral surface subjected to constant heat flow (*Q*), it is possible to calculate the parameter *m* defined as [33]:

(2) $$m=\frac{T(t)-T_{i}}{\sqrt{t}}=\frac{2\cdot Q}{\sqrt{\pi }}\cdot {\left(\sqrt{k\cdot \rho \cdot C_{p}}\right)}^{-1}$$

where, *k* is thermal conductivity, *ρ* is the density, and *Cp* is the specific heat at constant pressure. The water content affects the thermal capacity of the materials [33]. The diffusion of water within the wood fibers increases the specific heat, conductivity, and density of the materials, so that such diffusion can be quantified using the *m* parameter defined in Equation (2).

The dependence of these three parameters on the water content (*W*) is expressed by Equations (3)–(5).

(3) $$C_{p}=\frac{C_{p_{d}}-WC_{p_{w}}}{1+W}$$

(4) $$K=\frac{K_{d}-WK_{w}}{1+W}$$

(5) $$\rho =\rho_{d}\left(1+W\right)$$

The expression of thermal conductivity, as Ludwig et al. said in Reference [33], can be assumed in analogy to the electrical conductivity. In fact, the Fourier law and the Ohm law present the same form, furthermore electrical and thermal conduction lay on the same physical phenomena.

In the case of a constant heating (*Q*) flux applied to the separation surface of a semi-infinite medium, the expression of the evolution of the surface temperature *T*, starting from the initial temperature *T*_0_, becomes the Equation (6).

(6) $$T=T_{0}+\frac{Q2\sqrt{t}}{\sqrt{\pi k\rho c_{p}}}$$

that leads to Equation (7).

(7) $$T-T_{0}=m\sqrt{t}$$

The abovementioned discussion explains how, taking into account Equation (7), Equation (2) can be obtained and, therefore, the *m* parameter can be defined via the definition of the thermal effusivity *e* represented by Equation (8).

(8) $$e=\sqrt{k\rho C_{p}}$$

**Figure 2:**
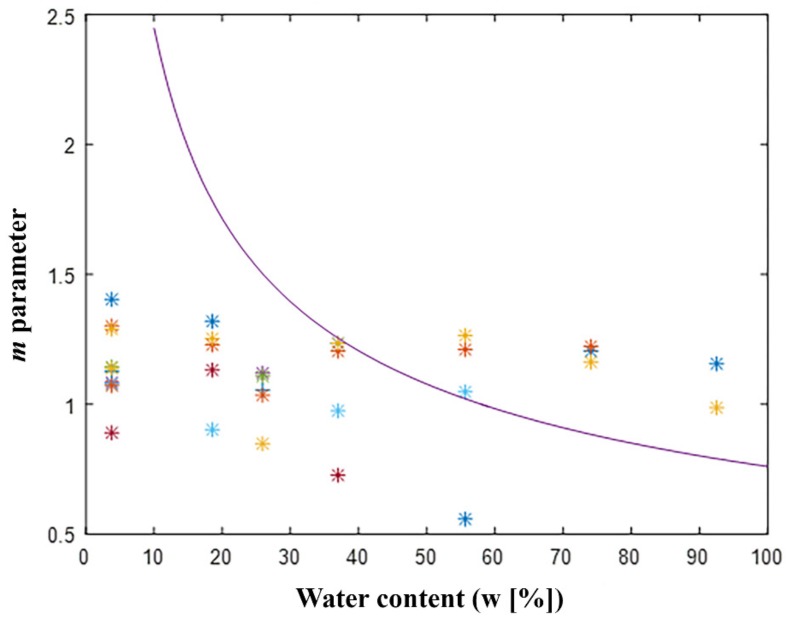
Correlation of results among the water content (w [%]) and the *m*-parameter.

Figure 2 shows the variation of the *m* parameter obtained from Equation (2); it is linked to the water content. Symbols having the same color and shape represent the behavior of a wooden sample for several water contents. The *m* parameter values obtained for seven samples subjected to twenty-seven different percentages of water content are shown in Figure 2. The solid line shows the theoretical result obtained from Equation (2) using the addiction to thermal conductivity, density, and specific heat at constant pressure with a content of water expressed in Equations (3)–(5). It should be noticed that the model (*m* parameter vs. water content) has previously been used by other researchers [33] which demonstrated the strong dependence to the type of wood under inspection.

In particular, it represents the best fit of a set of experimental data obtained with Equation (9). From a physical point-of-view, it is the angular coefficient of the temperature increase in active thermal tests on wooden samples with different water content. Equation (9) comes from Equations (3)–(5) and (8) [33].

(9) $$e=\frac{1}{1+W}\sqrt{\left(k_{w}\rho C_{p_{w}}\right)W^{3}+\left(2k_{w}\rho C_{p}+k\rho C_{p_{w}}\right)W^{2}+\left(k_{w}\rho C_{p}+k\rho C_{p}+k\rho C_{w}\right)W+k\rho C_{p}}$$

In our case, the model (*m* parameter vs. water content) appears reasonable for high water contents (w [%]) while, for low water content, it does not solve the aforementioned dependence.

The thermal effusivity *e* plays an important role in the definition of the *m* parameter since it is multiplied by $\sqrt{t}$ in the denominator of Equation (2). Thermal effusivity should increase with water content. This can be explained by the fact that, in dry state, in absence of interstitial water, heat transfer is essentially provided by fibers. In case of water content increases, heat transfer is mainly provided by interstitial water in material.

It should be noted that, at the critical moisture content for wood preservation (from 12% to 25%), the values of evaporation flux are very low (<10^−5^ kg/m^2^ s). Therefore, the cooling down due to evaporation produces so low signal (~0.5 °C) that it can be masked by other thermal effects.

For this reason, the preliminary drying of the samples may help to calculate the *m* parameter and, therefore, improve the preliminary results here shown. The samples should be firstly drying in oven at 103 °C until constant mass is obtained [34], and secondly should then kept in aluminium foil and in a plastic casing to attain hygrothermal equilibrium with the atmosphere, after a period of 48 h [35].

### 3.2. Speckle Pattern Method

Different contrast techniques [36] can be used to determining the water content using a speckle pattern. In this work, the photothermal speckle modulation (PSM) technique was adopted [37]. A sequence of 60 specklegrams acquired in 600 s was collected per water diffusion test.

**Figure 3:**
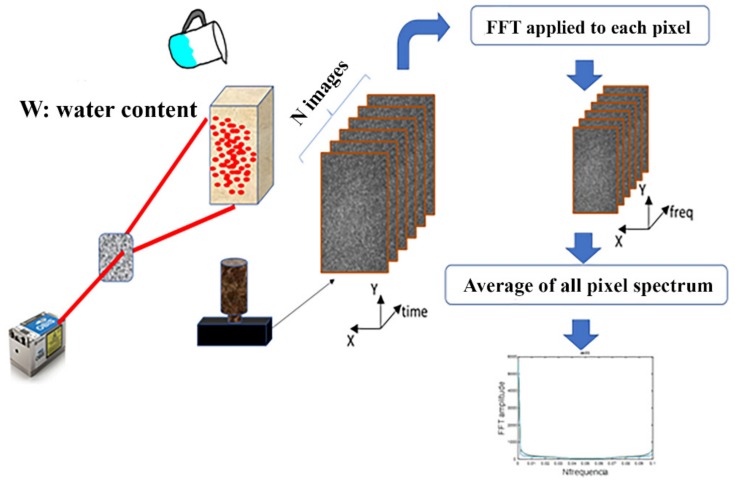
Schema to obtain the photothermal speckle modulation (PSM) parameter.

The PSM parameter was obtained from the specklegrams sequence according to the diagram presented in Figure 3 as follows:

1. The Fast Fourier Transform (FFT) was applied to each pixel of the sequence individually,
2. The FFT spectrum for each pixel was averaged with all pixels resulting in the PSM spectrum of the specklegram sequence.

**Figure 4:**
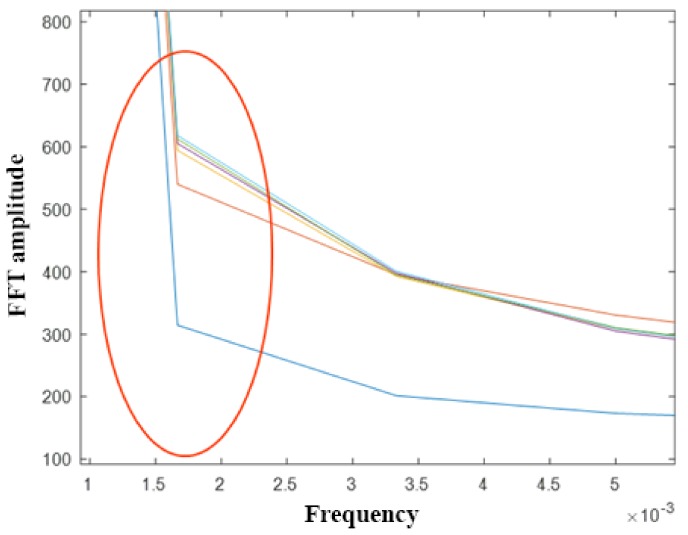
PSM parameter (amplitude of the first frequency of the averaged spectrum).

The reader should notice that the PSM spectrum is of interest at low-frequency values because water diffusion is slow. The lowest frequency value obtained can be correlated with water diffusion. Figure 4 shows how this variation occurs when the water content changes. The 1/600 Hz frequency component was the selected one. This frequency can be lowered by increasing the view window.

**Figure 5:**
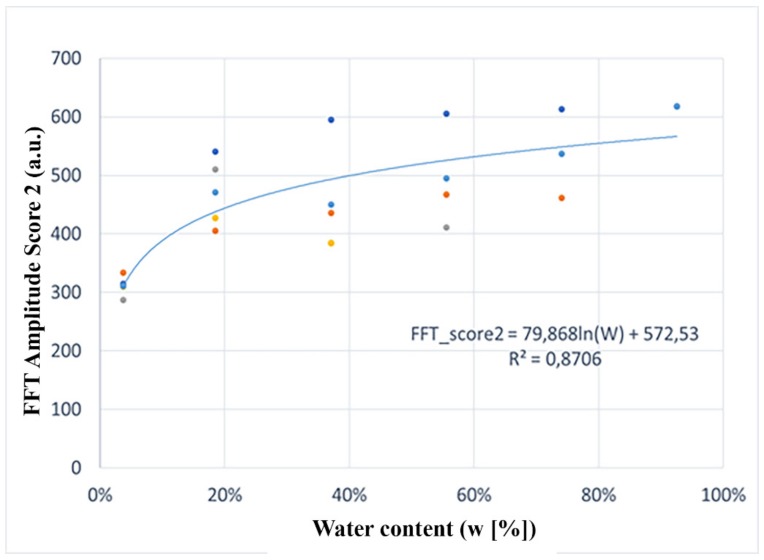
PSM parameter (amplitude of the second frequency of the averaged spectrum) vs. water content.

Variations in the FFT score of the second frequency are presented in Figure 5. They are related to the water content in eight samples inspected. The results allow the interpolation with a logarithmic nonlinear function shown in Equation (10) that links them to the water content by using an adjustment factor (i.e., R^2^ =0.8706).

(10) $$FFT_{score2}=79.868\cdot \ln\left(W\right)+572.53$$

Looking at the results, it is possible to say that the PSM model (FFT Amplitude score–2nd frequency vs. water content) is robust for both high and low water contents (w[%] in wooden samples).

Therefore, the advantages in the using of a speckle pattern are threefold: a) it do not need of any heating source, b) being very similar to electronic speckle pattern interferometry (ESPI), it can detect displacements on a surface of less than the wavelength of the illuminating coherent light source [38], and c) although the drying procedure explained above is highly desirable, the samples do not need a special preparation before analysis.

## 4. Conclusions

In this work, an integrated study among two NDT techniques able to measure the water content (w [%]) in hexahedral wooden specimens is presented. The method based on active IRT in the SH approach allowed the calculation of the *m* parameter related to the thermophysical properties of density, thermal conductivity, and specific heat at constant pressure. Results appear reasonable for high water contents (w [%]), while for low water contents, more research is needed in order to obtain a good agreement to the theoretical values.

Regarding the experimental setup, it can be improved by placing a glass filter between the lamp and the specimen during the heating-up phase in order to minimize the noise due to the emission of the bulbs when exceeding the 100 °C [33].

Instead, in the speckle interferometry method, the PSM technique was used for the first time to inspect the behavior of wooden samples subject to moisture. Although it is based on a nonlinear behavior, the model appears robust for both high and low water contents introduced into the sample under analysis. The spectral components of speckle pattern offer information about the water content. The water flow modified the score of lower frequencies of the speckle patterns. The lowest frequency represented in the spectrum is related to the size of the time window of the recorded data. Indeed, to a larger window corresponds a lower frequency; therefore, a larger window could offer better results. The window used herein was 30 min.

At this point it is possible to say that, whether combined with each other, such techniques can be able to provide important information concerning the status of degradation of wooden structures belonging to civil engineering and restoration fields [39,40]. Future development of the work could be the use of ultrasound thermography [41], with the aim to understand the “impact” of water on such type of wooden materials, i.e., to retrieve the depth’s impact on water content measurement [42].

Finally, since the results are not conclusive for the IRT testing setup inherent to the different water contents (which indicates that the *m* factor might need additional research and tuning), a good idea to studying more in the future this research area may be the use of a calibration step along with the imaging (or mapping) of the results.
